# Supplementary material for: Mechanisms governing the pioneering and redistribution capabilities of the non-classical pioneer PU.1
Source: Nat Commun. 2020 Jan 21;11:402. doi: 10.1038/s41467-019-13960-2 (PMC6972792; doi:10.1038/s41467-019-13960-2)
Supplement: Supplementary file 7 — Source data [file 41467_2019_13960_MOESM7_ESM.zip › Source_Data/FigureS2/FigureS2B_MotifScanOutput/KmeansCluster4/homerResults/motif26.similar.html]

motif26

## Information for motif26

T
C
A
G
G
T
A
C
T
C
G
A
C
G
A
T
C
T
A
G
C
T
A
G
C
T
A
G
C
T
A
G
C
T
A
G
G
T
C
A
C
G
T
A
G
T
C
A
A
G
C
T
  
Reverse Opposite:  

C
T
G
A
A
C
G
T
C
G
A
T
A
C
G
T
A
G
T
C
A
G
T
C
A
G
T
C
G
A
T
C
G
A
T
C
C
G
T
A
A
C
G
T
A
C
T
G
A
G
T
C
  

|  |  |
| --- | --- |
| p-value: | 1e-42 |
| log p-value: | -9.779e+01 |
| Information Content per bp: | 1.788 |
| Number of Target Sequences with motif | 87.0 |
| Percentage of Target Sequences with motif | 1.93% |
| Number of Background Sequences with motif | 81.5 |
| Percentage of Background Sequences with motif | 0.20% |
| Average Position of motif in Targets | 114.5 +/- 58.3bp |
| Average Position of motif in Background | 80.0 +/- 51.3bp |
| Strand Bias (log2 ratio + to - strand density) | 0.5 |
| Multiplicity (# of sites on avg that occur together) | 1.00 |
| Motif File: | file (matrix) reverse opposite |

### Similar de novo motifs found

|  |  |  |  |  |  |  |  |
| --- | --- | --- | --- | --- | --- | --- | --- |
| Rank | Match Score | Redundant Motif | P-value | log P-value | % of Targets | % of Background | Motif file |
| 1 | 0.808 | C T A G A C G T C G A T A C G T A G T C A G T C A G T C G T A C G T A C G T C A A G C T | 1e-23 | -54.585453 | 1.66% | 0.32% | motif file (matrix) |
| 2 | 0.901 | T A C G A C G T A C G T A C G T A G T C A G T C A G T C G T A C A G T C C G T A G A C T C T A G | 1e-21 | -50.084870 | 0.93% | 0.09% | motif file (matrix) |
